# Biofilm formation following chitosan-based varnish or chlorhexidine-fluoride varnish application in patients undergoing fixed orthodontic treatment: a double blinded randomised controlled trial

**DOI:** 10.1186/s12903-021-01805-8

**Published:** 2021-09-23

**Authors:** Preethi Poornima, Jogikalmat Krithikadatta, Ratna Rachel Ponraj, Natanasabapathy Velmurugan, Anil Kishen

**Affiliations:** 1 Department of Conservative Dentistry and Endodontics, Faculty of Dentistry, MAHER, Chennai, India; 2 Department of Cariology, Saveetha Dental College and Hospitals, Chennai, India; 3 Department of Orthodontics, Manipal Medical College, Melaka, Malaysia; 4 Faculty of Dentistry, University of Toronto, Toronto, ON Canada

**Keywords:** Dental plaque, Chitosan, Chlorhexidene, Fixed orthodontic therapy, Bonded Bracket Index

## Abstract

**Background:**

Orthodontic treatment poses an increased risk of plaque accumulation and demineralisation of enamel leading to white spot lesion around the brackets. This parallel arm trial aims to assess the degree of bacterial plaque formation adjacent to orthodontic brackets, following the application of a chitosan-based varnish or chlorhexidene-fluoride varnish.

**Methods:**

A total of 200 teeth from 20 patients undergoing fixed orthodontic therapy were assessed and biofilm formation around the brackets were recorded using the Bonded Bracket Index (Plaque index) at baseline and weekly for 6 weeks. The bacterial count and plaque pH at corresponding weekly intervals were also recorded. Following bracket bonding, the patients were cluster randomised to receive chitosan-based varnish-CHS (UNO Gel Bioschell, Germiphene corp., Brantford, Canada) or chlorhexidine-fluoride varnish-CFV (Cervitec F, Ivoclar Vivadent, Schaan, Liechtenstein) every week on the representative teeth respectively. BBI proportions were compared between groups at all time intervals using Chi square test. Mean plaque bacterial count and plaque pH were compared using Mann Whitney U test and Tukey’s HSD test respectively.

**Results:**

Baseline characteristics were similar between the groups: Mean age was CHS = 23 and CFV = 21; male to female ratio was CHS = 5/5, CFV = 7/3. At the end of 6 weeks, chitosan-based varnish performed equal to chlorhexidine-fluoride varnish (*P* > 0.05) with 98% and 95% of teeth with acceptable scores respectively. The plaque bacterial count significantly reduced at 6 weeks for both varnish compared to the baseline; The value for CHS was 0.43 ± 0.4 × 10^4^ and CFV was 0.77 ± 0.64 × 10^4^ CFU (*P* < 0.05), with no difference between both the varnishes. Both varnishes had no effect on the plaque pH that remained neutral.

**Conclusion:**

This trial showed that both chitosan-based varnish and chlorhexidine-fluoride varnish reduced bacterial count, while the plaque pH remained neutral over a period of six weeks in patients undergoing fixed orthodontic therapy. The anti-plaque effects of the natural biopolymeric chitosan-based varnish was similar to that of chlorhexidine-fluoride varnish, a known chemotherapeutic agent.

*Registration*: This trial protocol was registered with https://www.ctri.nic.in (CTRI/2019/05/018896). (Date of registration 02/05/2019).

*Protocol*: The protocol was not published before trial commencement.

## Introduction

Orthodontic fixed appliance therapy is the most preferred mode of treatment for most type of malocclusions [1]. There is a rapid shift in the bacterial flora of dental plaque following bracket placement. The high levels of bacterial plaque formed around the bracket are capable of decreasing the pH of plaque in orthodontic patients [2]. The most common sites for plaque formation and bacterial adhesion are at the bracket, adhesive and enamel surfaces [3]. The quality and quantity of plaque accumulated depends upon several factors such as design, surface characteristics, roughness, free energy of the brackets as well as the composite resin characteristics [4, 5]. Co-existence of these factors are essential for the development of white spot lesion (WSL) [6]. The prolonged plaque accumulation at the bracket-tooth interface in turn leads to decrease in the pH that tips the demineralization-remineralisation balance toward mineral loss [7]. WSLs are noticeable around the brackets within 1 month of bracket placement, although the formation of caries lesion typically requires at least 6 months. These lesions are commonly seen on the buccal surfaces of teeth around the brackets, especially in the cervical region [8].

*Streptococcus mutans* has been implicated as the principle etiological factor in the development of dental caries due to their aciduric and acidogenic properties, as well as its ability to rapidly adhere and accumulate on the tooth surface [9]. It has been reported that the extracellular matrix namely water-insoluble glucan synthesized by *S. mutans* contribute to the structural stability, and integrity of dental plaque. Furthermore, the plaque extracellular matrix allows bacteria to adhere to tooth surfaces besides protecting the bacteria against noxious stimuli and other environmental threats [10].

The elimination of plaque is considered as an important therapeutic strategy to prevent WSL [11]. Previous studies have investigated the most appropriate plaque elimination strategy for orthodontic patients, including electric tooth brushes, mouth washes and tooth pastes for plaque elimination [12–15]. Apart from the routine oral hygiene measures, other preventive measures includes; chemo-prophylactic methods such as use of fluoride varnish, chlorhexidine, xylitol, antimicrobials, calcium-containing remineralisation products that can help prevent enamel demineralization, enhance remineralisation, and modify patient and biofilm factors. Chlorhexidine is a cationic bis- biguanide that exhibits both bacteriostatic and bactericidal effect, which depends upon its concentration and has been considered as the gold standard antibacterial agent [16]. Concomitantly, fluoride varnishes, apart from inhibiting the metabolic activity of plaque bacteria [17], also remineralises the enamel surface and renders the enamel resistant to acid by fluorapatite formation [18]. However, there has been certain global concerns on fluoride applications. Fluoride uptake occurs both systemically [19] and topically via different methods. Thus it is difficult to monitor the degree of fluoride exposure in an individual as a function of time [20]. Recent Canadian birth cohort studies have established the association of fluoride with lower IQ among children exposed to environmental fluorides [21]. With fluoride being classified as a neurotoxin [22] there has been increasing concerns amongst parents over fluoride preventive strategies [23, 24]; compelling research for alternative caries prevention methods.

Chitosan, a natural biopolymer of marine origin has recently attracted attention due to its significant antimicrobial properties and advantages of being nontoxic, biodegradable and biocompatible. Chitosan is a derivative of chitin which contains poly(1,4-b-D-glucopyranosamine). When chitosan molecules are been subjected to methylation process, as a result of quaternization of the amino groups a positively charged salt of Trimethyl chitosan is formed [25]. The electrostatic interaction between positively charged chitosan sites and negatively charged microbial cell membranes is responsible for lysis. Mode of action of chitosan as a cationic biocide is by adsorption of microbial cell, diffusion through the cell wall, adsorption and destruction of plasma membrane, cytoplasmic membrane leakage and cell death [26]. As nano particles of chitosan has higher penetration rate. In vitro studies were carried out to study nano chitosan inhibition capability by measuring cell viability, remaining biofilm mass and biofilm mass reduction in dual species biofilm treated with various concentrations of nano chitosan [27]. The gel of chitosan was achieved through polymer dilution in acetic acid and has been suggested as a preventive therapeutic material against dental caries. It has shown to exhibit a broad antibacterial, anti-adherence and anti-biofilm characteristics [28]. Chitosan-based varnish is found to be effective in treating dentine hypersensitivity [29]. The ability of this varnish to limit oral biofilm formation has not been tested clinically. This formulation does not involve any chemicals other than a bioinert vehicle, thus improves the physical properties of the formulation. The purpose of this study was to assess the inhibition of biofilm formation following the application of a biopolymeric chitosan varnish or chlorhexidine-fluoride based varnish in patients undergoing fixed orthodontic therapy.

## Materials and methods

### Trial design and any changes after trial commencement

This was a parallel arm, randomised controlled trial with 1:1 allocation ratio.

### Participants, eligibility criteria, and setting

Patients scheduled for comprehensive orthodontic treatment at the Department of Orthodontics of Faculty of Dentistry, Meenakshi Academy of Higher Education and Research (MAHER) University were invited to participate in the study. The study was approved by the ethical committee of MAHER University (IEC Ref No. MADC/IEC/015/2017). This trial protocol was registered with Clinical Trial Registry of India (www.ctri.nic.in) CTRI/2019/05/018896.

Male and female patients undergoing fixed orthodontic therapy with conventional metal brackets, involving intact maxillary arch with permanent dentition between the age group of 16–32 years were included in the trial. Patients with good oral hygiene and with no incipient lesion were included. Exclusion criteria for the trial was patients requiring metal self-ligating or ceramic brackets and other fixed/removable appliances. Patients with periodontitis with probing depth > 4 mm, systemic diseases, fluorosis or antibiotics use 3 months prior to the study, smokers, pregnant ladies, patients with cleft lip/cleft palate and other dento-facial abnormalities were excluded from the study.

### Sample size calculation

The sample size for this parallel arm randomised controlled trial was calculated based on the pilot trial involving 30 teeth per group (n = 3 patients), for a power of 85%, alpha error at 5% and with an effect size of 0.46; the total number of teeth per group was 85 teeth. A dropout rate of 10% was added and sample size was rounded off to include 100 teeth (10 patients) per group with an allocation ratio of 1:1.

### Interventions

A week before bracket placement, oral hygiene instructions were reinforced to all the patients and modified Bass technique of brushing was demonstrated. All the patients received professional oral prophylaxis one week prior to the study.

#### Bonded bracket plaque index

It is primarily a plaque scoring index, developed to determine the amount of microbial plaque accumulation on teeth with brackets. A single pre-calibrated trained clinician performed all the clinical examination and sample collection. Among them, 20% of patients were screened again by the principal investigator and inter-evaluator agreement was calculated. BBI was recorded from maxillary right second premolar to maxillary left second premolar (n = 10 teeth, per patient) according to the following scores [30].

1. No plaque on the bracket or on the tooth surface
2. Plaque only on the orthodontic bracket
3. Microbial plaque on the bracket and tooth surface but not spreading towards the gingiva
4. Microbial plaque on the bracket and tooth surface spreading towards the papilla
5. Microbial plaque on the bracket and tooth surface and part of gingiva is covered with plaque
6. Microbial plaque on the bracket and tooth surface and part of gingiva is totally covered with plaque

#### Biofilm sample collection method and processing

Baseline data for Plaque Index, plaque pH and plaque bacterial count were tabulated as (T0) from the representative teeth. The score was taken at all time intervals for 6 weeks (T1–T6) and were recorded every week following bracket placement.

#### Plaque pH

All appointments were fixed in the morning between 8 and 9am. The patients were asked to refrain from brushing for 24 h and were asked not to eat until completion of sample collection. Following isolation of the labial maxillary surfaces with cotton rolls, pooled plaque samples were obtained from the labial surface of the maxillary right and left lateral incisor and canine to be bracketed. The plaque samples weighing ≈ 2 mg were collected with a sterile E2 Hu-Friedy spoon excavator (Hu-Friedy Mfg. Co., Chicago, USA) and transferred to 5 ml of double distilled deionised water. The resting pH was then recorded within 90 s with a digital pH meter (µ pH System 361, Systronics India Pvt. Ltd. India). The pH meter was calibrated with standard buffers of pH 4 and 7 before recording the plaque pH.

#### Plaque bacterial count

Pooled plaque sample obtained from the buccal surface of maxillary right and left first and second premolar (as described above) to be bracketed were transferred into 2 ml eppendorf tubes containing BHI broth (Hi-Media Laboratories, Mumbai, India) for bacterial culturing. The samples were homogenised by vortexing for 5 min, 1 ml of samples was diluted from 1:10 to 1:10^6.^ After dilution 0.1 ml of the diluted samples were carried and spread over agar plates for microbial growth. The agar plates were incubated at 37 °C, for 48 h in aerobic condition supplemented with 5% carbon dioxide. Bacterial colonies were morphologically identified and counted as Colony Forming Units (CFU) using a colony counting grid (Hi-Media Laboratories, Mumbai, India).

#### Bracket bonding procedure

Once the baseline data was recorded the maxillary arch was bonded with conventional stainless-steel brackets (Stainless steel Bracket-Mini, Ormco, California, USA). The bonding procedure was done as follows; all buccal/labial surface of the teeth to be bonded was polished with a rubber cup using pumice with a slow speed hand piece (Contra angle FX22, NSK Confident Sales India Pvt. Ltd). Then the teeth were rinsed with water, air dried and etched with 37% orthophosphoric acid (d-tech, D Tech Dental Technology, Pune, India) for 30 s. The acid was rinsed off and the teeth dried until the enamel exhibited a frosted appearance. Transbond XT primer (3 M Unitek, Monrovia, CA, USA) was applied on the etched enamel surface and air thinned. Finally, “0.022” edgewise brackets was placed on the teeth with an appropriate amount of Transbond XT applied on the bracket base. Excessive adhesive around the bracket was removed and light cured for 15 s. After bracket bonding, comprehensive oral hygiene instructions were given to the patients.

#### Varnish application

Following bracket placement varnish application procedure was performed on right maxillary premolar to the left maxillary premolar (n = 10 teeth/patient). Prior to the application of the varnish, oral prophylaxis was carried out for the teeth at every visit. The representative teeth were then isolated with cotton rolls, saliva ejector and dried with a gentle blow of air for 30 s using 3-way air syringe. One drop of the designated varnish was dispensed into a dappen dish and applied around the bracket using a microbrush (Micro Brush Applicator, 3MESPE St. Paul, Minnesota, USA). The varnish was allowed to dry for 1 min. The patients were instructed not to rinse and refrain from eating or drinking for 1 h after the application of the varnish. The same varnish application protocol was followed at every week for 6 weeks for assessment in both the groups.

### Outcomes (primary and secondary) and any changes after trial commencement

Biofilm formation using Bonded Bracket Index (Plaque index) following the use of varnish was the primary outcome. Secondary objectives assessed were the bacterial count and estimate the plaque pH following the application of the varnishes. There were no changes in the outcome after the trial commencement.

### Interim analyses and stopping guidelines

Not applicable.

### Randomization (random number generation, allocation concealment, implementation)

Computer generated (Minitab Statistical Software, Pennsylvania, USA) cluster randomisation was used to allot the 20 patients to their respective groups, received either Chitosan-based varnish (CHS) (UNO Gel Bioschell, Germiphene company, Brantford, Canada Chlorhexidine-flouride varnish (CFV) ((Cervitec F, Ivoclar Vivadent, Schaan, Liechtenstein). The allocation of patients to both interventions was concealed using brown opaque envelopes and opened prior to application. The enrolment of patients and the implementation of randomisation was performed by the nursing assistant who was not a part of the study.

### Blinding

The operators could not be blinded because of the distinct differences in the consistency of the varnishes. However, outcome assessor, patients and principle investigator remained blinded to the study.

### Data collection

Data was collected and recorded in an Excel sheet (Microsoft Office 2010, version 14.0, Microsoft Corporation, Washington, USA). An intention-to-treat analysis was performed. BBI scores 0,1 are clinically accepted and were combined as acceptable plaque levels because the plaque does not extend to the tooth surface. BBI scores above 2 were combined together as clinically unacceptable scores. While integrating scores per patient for BBI, a score on any one tooth of 2 or more was considered unacceptable.

### Statistical analysis (primary and secondary outcomes, subgroup analyses)

The statistical analysis was conducted at 2 levels for the primary objective (BBI)-tooth level and patient level. The influence of a patient on the cluster of teeth was determined in the patient level analysis of BBI score. All 3 objectives were analyzed for inter-group and intra-group comparisons at all time points. The grouped BBI data based on cut-off points were subjected to Chi square test and Fischer's exact test. One-Sample Kolmogorov–Smirnov test was performed to assess normal distribution of Plaque pH and plaque bacterial count data. Based on the distribution, an independent t-test and Mann Whitney-U test was performed. P value was set as less than 0.5. The data was analyzed using SPSS software (SPSS Inc. Version 17, Chicago, USA).

## Results

### Base line data

**Fig. 1:**
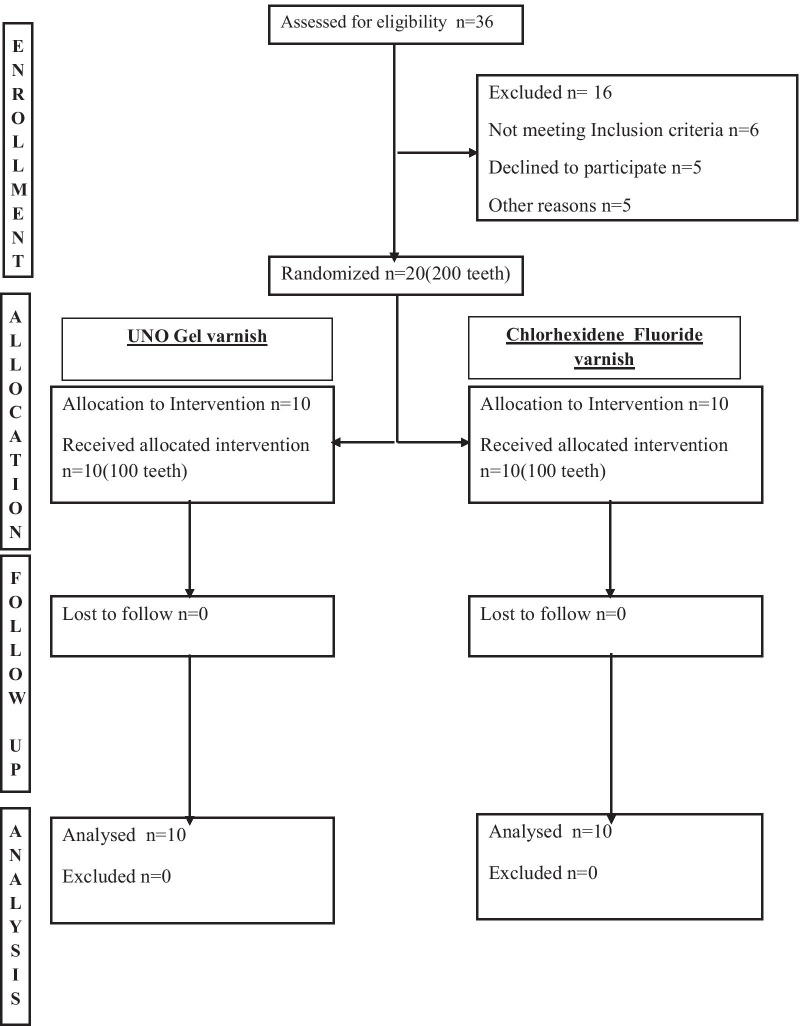
CONSORT flow chart for the trial

A total of 200 teeth from 20 patients were assessed, mean age and standard deviation of the patients were CHS = 23 ± 4.7, CFV = 21 ± 3 with *p*-Value 0.197, and male to female ratios were CHS = 5/5, CFV = 7/3) were randomised in 1:1 ratio to either of the varnishes. No patients were lost to follow-up (Fig. 1). The patients were recruited from the month of May 2019 to July 2019 and were followed up for a period of six weeks. The baseline demographic details of participants recruited in the trial are given in (Table 1). The variables showed no statistically significant differences at baseline. The proportion of acceptable score was higher in both the groups.

**Table 1:** Baseline demographic data of participants recruited in trial with chitosan-based varnish and chlorhexidine-fluoride varnish

Parameter	Chitosan-based varnish (CHS)	Chlorhexidine-fluoride varnish (CFV)	*p*-value
*Age*			
Mean ± standard deviation	23.3 ± 4.7	21 ± 3.1	0.197
*Gender*			
Male/female	5/5	7/3	0.650
*Bonded Bracket Index*			
Acceptable	95 (9)	97 (9)	0.360
Unacceptable	5 (1)	3 (1)	
*Bacterial count*			
Mean ± standard deviation (× 10^4^)	3.6 ± 3.9	4.4 ± 3.8	0.641
*Plaque pH*			
Mean ± standard deviation	6.3 ± 0.97	6.2 ± 0.61	0.537

(−) denotes patient level estimates

### Numbers analyzed for each outcome, estimation and precision, subgroup analyses

Bonded Bracket Index (BBI) (n = 200 teeth), plaque bacterial count (n = 80 teeth) and plaque pH (n = 80 teeth) were recorded. Grouped BBI scores were similar in both the groups with no significant difference at all time points for both tooth-level and patient-level comparisons (Table 2). The data on Plaque pH was normally distributed while that on plaque bacterial count did not follow normal distribution. Mann Whitney-U test for plaque bacterial counts showed insignificant differences between CHS and CFV groups at all time intervals for tooth-level (Table 3). The plaque bacterial count significantly reduced at 6 weeks for both varnish compared to baseline; CHS-0.43 ± 0.4 × 10^4^ and CFV-0.77 ± 0.64 × 10^4^ CFU (*P* < 0.05), with no difference between both the varnishes. Similarly, Turkey’s HSD showed no differences with plaque pH values between groups for tooth-level comparisons (Table 4). CONSORT flow chart is described in Fig. 1. There was no adverse reaction noted with both interventions.

**Table 2:** BBI index scores at different time intervals with chitosan-based varnish and chlorhexidine-fluoride varnish

Time interval	Groups		Total	*p*-value
	Chitosan-based varnish (CHS)	Chlorhexidine-fluoride varnish (CFV)		
*T0*				
Acceptable	95 (9)	97 (9)	192 (18)	0.36 (1)
Unacceptable	5 (1)	3 (1)	8 (2)	
*T1*				
Acceptable	87 (4)	92 (5)	179 (9)	0.18 (0.7)
Unacceptable	13 (6)	8 (5)	21 (11)	
*T2*				
Acceptable	92 (5)	94 (9)	186 (14)	0.4 (0.05)
Unacceptable	8 (5)	6 (1)	14 (6)	
*T3*				
Acceptable	91 (6)	89 (5)	180 (11)	0.41 (0.07)
Unacceptable	9 (4)	11 (5)	20 (9)	
*T4*				
Acceptable	94 (8)	96 (8)	190 (16)	0.37 (1)
Unacceptable	6 (2)	4 (2)	10 (4)	
*T5*				
Acceptable	90 (7)	95 (8)	185 (15)	0.14 (0.6)
Unacceptable	10 (3)	5 (2)	15 (5)	
*T6*				
Acceptable	98 (10)	95 (8)	193 (18)	0.22 (0.5)
Unacceptable	2 (0)	5 (2)	7 (2)	

Acceptable, BBI scores 0,1; Unacceptable, BBI scores 2,3,4

(−) denotes patient level estimates

**Table 3:** Mean and standard deviation of plaque bacterial count at baseline and different time intervals

Time interval	Chitosan-based varnish (CHS)			Chlorhexidine-fluoride varnish (CFV)		
	n (teeth)	Mean ± standard deviation (× 10^4^)	Median (× 10^4^)	n (teeth)	Mean ± standard deviation (× 10^4^)	Median (× 10^4^)
BCT0	40	3.6 ± 3.9	2.3	40	4.4 ± 3.8	4.5
BCT1	40	1.6 ± 1.3	1.5	40	2.2 ± 2.1	1.0
BCT2	40	1.1 ± 1.0	1.0	40	1.2 ± 1.5	0.75
BCT3	40	0.88 ± 1.0	1.0	40	2.4 ± 2.9	1.0
BCT4	40	1.3 ± 0.55	0.55	40	1.5 ± 1.7	1.0
BCT5*	40	0.44 ± 0.1	0.1	40	0.88 ± 0.77	0.75
BCT6*	40	0.43 ± 0.4	0.4	40	0.77 ± 0.64	0.6

BC, bacterial count

*Significant reduction in bacterial colonies compared to baseline

**Table 4:** Mean and standard deviation of plaque pH with 95% confidence interval at baseline and at different time intervals

Time interval	Chitosan-based varnish (CHS)				Chlorhexidine-fluoride varnish (CFV)			
	n (teeth)	Mean ± standard deviation	95% Confidence interval		n (teeth)	Mean ± standard deviation	95% Confidence interval	
			Lower bound	Upper bound			Lower bound	Upper bound
PPHT0	40	6.3 ± 0.97	5.8	6.8	40	6.2 ± 0.61	6.0	6.5
PPHT1	40	6.2 ± 0.72	5.8	6.5	40	6.03 ± 0.84	5.6	6.4
PPHT2	40	6.2 ± 0.74	5.8	6.5	40	6.10 ± 0.56	5.9	6.3
PPHT3	40	6.1 ± 0.51	5.9	6.4	40	6.2 ± 0.56	6.0	6.5
PPHT4	40	6.1 ± 0.90	5.7	6.6	40	6.0 ± 0.59	5.7	6.2
PPHT5	40	6.2 ± 0.67	5.9	6.5	40	6.2 ± 0.46	6.0	6.4
PPHT6	40	6.4 ± 0.62	6.1	6.7	40	6.5 ± 0.42	6.3	6.7

PPH, Plaque Ph

## Discussion

Caries prevention in fixed orthodontic therapy poses a significant challenge, since bonding of brackets creates a high caries risk environment [31]. WSL is a common complication of fixed orthodontic therapy especially on the maxillary anterior teeth with a prevalence as high as 50% [32]. The most commonly affected teeth in order of severity are maxillary lateral incisor followed by maxillary canine, premolars and others (17–34%) [33]. The quality of plaque, its bacterial contents and the pH have a direct effect on the cariogenic potential of dental plaque formed around brackets [34]. A systematic review showed that the application of chlorhexidene varnish resulted in effective plaque control, decreased *S. mutans* count and reduced WSL in patients undergoing fixed orthodontic therapy [35]. It was also highlighted that a periodic application of fluoride varnish in patients undergoing fixed orthodontic treatment provided protection against WSL [32, 36]. Hence chlorhexidine-fluoride varnish was chosen as a standard of care in this trial. A weekly application of both varnishes was preferred over other time intervals as the frequency of application influenced the degree of plaque growth and bacterial inhibition [37, 38]. Chlorhexidine also displays adsorption and sustain release characteristics. Hence a cluster randomization was preferred in this trial over simple randomization or split mouth design. This eliminates the possibility of intervention contamination. In order to understand the influence of the individual patient behaviour on the cluster samples, the BBI scores were assessed at the tooth and patient levels. Insignificant differences at both levels eliminated the influence of outliers. Since the varnish was applied weekly, the teeth samples had no correlation between the time intervals. Thus a Chi-square test was used to assess the data between time intervals instead of the conventional generalised estimating equations.

Glucosyltransferase (Gtf) secreted by *S. mutans* can bind to the pellicle on the tooth surface and produce glucans for bacteria colonization and subsequent biofilm formation [39]. Hence chemotherapeutic agents aimed at interrupting bacterial colonization and extracellular polysaccharide (EPS) synthesis by Gtf have a promising approach towards oral plaque control [40]. One such natural biopolymer, which possess remarkable anti-biofilm properties is chitosan [41]. The ability of chitosan to inhibit biofilm depends on molecular weight, degree of deacetylation, concentration, exposure time and phase of biofilm development [42]. Costa et al. demonstrated that chitosan was capable of inhibiting biofilm formation for up to a period of 1 week, independent of its molecular weight. In addition, chitosan treatment resulted in significant reduction in the surviving bacteria found within the mature biofilm [43]. According to a systematic review of recent clinical studies, by Marco Cicciu et al. the use of chitosan has shown better reduction in bacterial biofilm when used in dental cements namely, Chitosan Modified Glass Ionomer Cements [44]. In another investigation, Arnaud et al. applied optical coherence tomography to highlight the penetration of chitosan and the mechanical barrier effect formed up to the dentinoenamel junction. This chitosan mediated enamel modification hindered acid penetration and subsequent enamel demineralisation [45]. Therefore, the potential of chitosan-based varnish to limit plaque formation around orthodontic brackets was worth exploring.

Acid production in cariogenic plaque is an important parameter in risk assessment and hence was studied in this trial [46]. The pH values measurements showed that the pH values for both the interventions were much above the critical pH of enamel and showed no significant difference between the two interventions. This could be attributed to the age of the plaque formed around the brackets and the presence of sucrose within the plaque [47, 48]. Since the plaque formed around the brackets were minimal in this trial, their immediate cariogenic potential could be questionable. We also believe that the methodology employed for plaque assessment in this study could have influenced the recorded values [49].

The current clinical trial demonstrated comparable plaque control effects of Chitosan varnish and chlorhexidine-fluoride varnish. The plaque inhibitory mechanism of chitosan can be explained by the alteration in the electrostatic interaction between the tooth pellicle surface and the bacterial cell. The positively charged chitosan chains attaches to the negatively charged cell surface. These chains of sufficient length forms bridges between the bacterial cells. Flocs are formed as soon as the bridges becomes effective, therefore inhibiting *S. mutans* colonization on the tooth surface [50, 51], and subsequent increase in the membrane permeability and leakage of intracellular material constituents, leading to cell death. The electrostatic interaction between chitosan and bacteria may also interfere with the mRNA synthesis and embedding protein synthesis [52]. Even sub-lethal concentration of chitosan is known to induce a successive decrease in cell wall hydrophobocity, altering the degree of bacterial adherence [51]. These effects were demonstrated by significant reduction in bacterial counts within the biofilm. The biopolymeric varnish consist of 0.1% chitosan nanoparticles dispersed in carboxymethyl solution. This was the minimal antibacterial concentration for the chitosan nanoparticles based on the degree of deacetylation [53] This concentration was also within the non-aggregating concentration to facilitate the application as a varnish [54]

The superior anti-plaque property of chlorhexidene is attributed to the three possible mechanism of action of chlorhexidene in inhibiting plaque are: (1) The blocking of acidic groups of salivary glycoproteins, which inhibits the formation of acquired pellicle. (2) The adsorption of chlorhexidene to the extracellular polysaccharides, resulting in reduced bacterial adherence. (3) Chlorhexidine may compete with the calcium ion agglutination factors of the plaque. Even low concentration of chlorhexidene (1%), exhibits bacteriostatic effect by interfering with the membrane transport allowing other light weight molecules to infiltrate into the microbial cells [55]. Chlorhexidene fluoride varnish used in this study contains 0.34% of chlorhexidene and 1400 ppm of aminofluoride that is 0.27% and cetylpyridinum chloride (0.5%) as active ingredients. These are cationic substances that adhere to surfaces of negatively charge cell walls of bacteria and thereby inhibit plaque formation and bacterial metabolism, fluoride also reduces the cariogenic lactic acid formation in plaque bacteria, and impairs bacterial glucose uptake and glycolysis [56]. Concomitantly, the use of fluoride and thymol along with chlorhexidene varnish was suggested to effectively inhibit plaque accumulation [57]. The combined effects of these active ingredients were demonstrated in this clinical trial. Nevertheless, the benefits of chlorhexidine components in a varnish has been debated [58].

Although chlorhexidene is a non-toxic agent, it has an unpleasant taste. It is known to alter the taste sensation, while affecting the mucous membranes and tongue [59]. Chlorhexidine can produce extrinsic staining of teeth, promote supragingival calculus formation and stain the margins of composite and glass ionomer restoration [60]. Recently, enhanced tolerance or even resistance to chlorhexidine has been reported in oral bacteria. When sub-inhibitory concentration of chlorhexidene is released in the oral cavity (a) the antimicrobial efficacy diminishes due to inactivation by salivary or serum proteins, and (b) bacterial tolerance increases due to the acquisition of a new plasmid in *S. mutans* genes [61]. Additionally, the concentration of fluoride in Chlorhexidine-fluoride Varnish is 1400 ppm is closer to that found in toothpaste [62]. This concentration is negligible for the application of caries prevention when compared to convention fluoride varnishes that usually contain 19,000–22,500 ppm. A recent clinical trial demonstrated that Cervitec with or without fluoride had similar effect in preventing plaque formation [63]. On similar lines, a systematic review has shown F to reduce the corrosion resistance of orthodontic brackets exposed to oral fluorides [64].

The current study demonstrated that chlorhexidine-fluoride varnish and chitosan varnish have similar effects of inhibiting plaque accumulation and reducing bacterial loads over a period of six weeks. The advantages of applying a natural biopolymer like chitosan could be considered as a better choice over the use of chemicals such as chlorhexidene or fluoride in inhibiting plaque formation in fixed orthodontic patients. The limitations of this trial are the short follow-up period and lack of information of cariogenic plaque. Extended follow-up and assessment of the incidence of WSL comparing these interventions and specific anti-cariogenic properties of chitosan varnish need to be explored. The results of this trial are generalisable to all patients undergoing fixed orthodontic therapy using conventional metal brackets and without additional appliances.

## Conclusion

This trial showed that both chitosan-based varnish and chlorhexidine-fluoride varnish reduced bacterial count, while the plaque pH remained neutral over a period of six weeks in patients undergoing fixed orthodontic therapy. The anti-plaque effects of the natural biopolymeric chitosan-based varnish was similar to that of chlorhexidine-fluoride varnish, a known chemotherapeutic agent.

## Data Availability

The datasets generated during and analyzed during the current study are not publicly available due to our university regulations, but are available from the corresponding author on reasonable request.
